# Species interactions in occurrence data for a community of tick-transmitted pathogens

**DOI:** 10.1038/sdata.2016.56

**Published:** 2016-08-01

**Authors:** Agustín Estrada-Peña, José de la Fuente

**Affiliations:** 1 Department of Animal Pathology, Faculty of Veterinary Medicine, University of Zaragoza, Zaragoza 50013-Zaragoza, Spain; 2 SaBio, Instituto de Investigación en Recursos Cinegéticos, IREC (CSIC, UCLM, JCCM), 13005 Ciudad Real, Spain; 3 Department of Veterinary Pathobiology, Center for Veterinary Health Sciences, Oklahoma State University, Stillwater, Oklahoma 74078, USA

**Keywords:** Ecological epidemiology, Lyme disease, Entomology, Microbial ecology

## Abstract

Interactions between tick species, their realized range of hosts, the pathogens they carry and transmit, and the geographic distribution of species in the Western Palearctic were determined based on evidence published between 1970–2014. These relationships were linked to remotely sensed features of temperature and vegetation and used to extract the network of interactions among the organisms. The resulting datasets focused on niche overlap among ticks and hosts, species interactions, and the fraction of the environmental niche in which tick-borne pathogens may circulate as a result of interactions and overlapping environmental traits. The resulting datasets provide a valuable resource for researchers interested in tick-borne pathogens, as they conciliate the abiotic and biotic sides of their niche, allowing exploration of the importance of each host species acting as a vertebrate reservoir in the circulation of tick-transmitted pathogens in the environmental niche.

## Background & Summary

Predicting which species occur together is one of the greatest challenges in ecology and requires a sound understanding of how the abiotic and biotic environments interact with dispersal processes across scales^1^, particularly when the possible spread of ticks and the (re)emergence of tick-transmitted pathogens are considered^2^. Using different methods, several studies have approached capturing the abiotic factor regulating the distribution of organisms, including parasitic arthropods, in what is commonly called ‘predictive mapping’^3–5^. In some cases, these studies are supported by interpolated climate datasets, which provide the abiotic side of the niche. These datasets are suitable for describing large climate patterns but unreliable for describing the abiotic niche of arthropods, as they may inflate the models^6^. In addition, though these datasets are excellent descriptors of Earth’s climate, they do not account for the many combinations of abiotic factors that may impact the communities of arthropod ectoparasites, such as ticks and their hosts^7^.

In the case of tick-transmitted pathogens, there is an increasing tendency to identify the abiotic niche of a tick by the territory in which the transmission of a pathogen could occur^8^, immediately associating the presence of the pathogen with the presence of the vector(s). However, this assumption is a translation of the multidimensional niche of the tick (described by a number of explanatory variables) into a map and the conjecture that pathogen reservoirs always overlap in the space with the tick to produce permanent foci of pathogen transmission. The fallacy of this argument is that it ignores that hosts also have a niche; therefore, the probability of a tick finding a host depends on both the host and tick density, disregarding the relative importance of a host in the transmission and reservoir capacity of a tick-transmitted pathogen. Advances in the last few years include broader knowledge of the relative contribution of some vertebrates to the circulation of tick-transmitted pathogens, with a focus on bacteria in the *Borrelia burgdorferi* complex^9^. These reports make important contributions to capturing the complexity of these communities of vectors, reservoirs, and pathogens, but they have a regional focus and report species-specific data rather than a community overview and cannot yet be translated into a modeling framework that simultaneously accounts for the abiotic and biotic components of these processes.

Here, we present a novel framework to scale from environmental traits, which have been classically suited to evaluate the risk of tick-transmitted pathogens, to the functioning of a community of pathogens. We aimed to formulate a synthesis of the relationships between hosts, ticks, and transmitted pathogens to (i) assess differing assumptions underlying the assembly of communities of pathogens; (ii) assess the relative importance of hypothesized drivers of diversity (classically, weather); and (iii) build a more predictive and dynamic framework for scaling from traits to the community of tick-transmitted pathogens. The methodology consolidates the diverging fields of biotic (reservoirs) and abiotic (climate) relationships between ticks into a single foundation. The paradigm is radically different from existing approaches^10^ because it describes these relationships in the dimensions of the environmental niche rather than its spatial projection. Such previous development^10^ provided only a basic set of records of ticks and their associated environmental traits, without further outcomes regarding the realized and predicted distribution of the hosts or the ecological relationships between ticks, hosts and pathogens. A complete set of environmental traits and the framework to evaluate rates of contact between partners and circulation of tick-borne pathogens were also lacking in existing publications^10^. In this new context, modelling outcomes do not result in maps as is the rule in the field, but in charts displaying the gradients of environmental variables. This is ecologically more reliable and provides a tractable background on which the impacts of climate trends can be evaluated, something that is unapproachable with current frameworks. We developed this workflow, providing the necessary climate datasets, realized and predicted distributions of ticks and hosts, and the interactions recorded between them, as well as scripts in a widely used programming environment for further calculations.

## Methods

**Figure 1:**
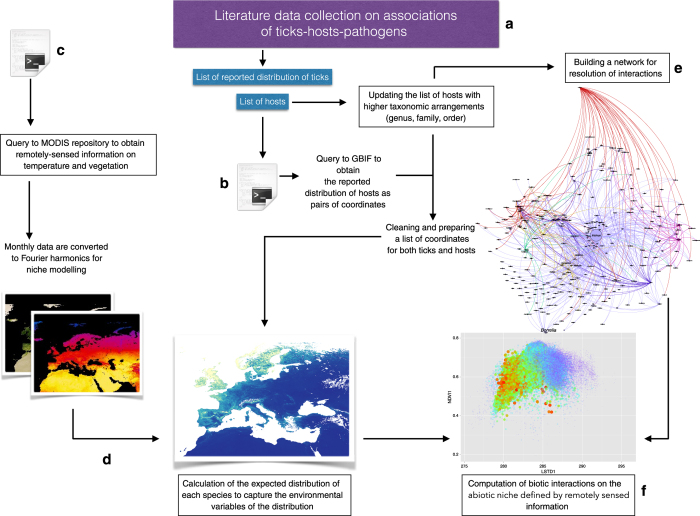
The data acquisition workflow. Data were acquired primarily from literature on the associations of ticks, hosts, and transmitted pathogens using several scripts prepared in the R programming environment to query data repositories (**a**). These queries locate and download the reported distribution of the vertebrates reported as hosts for ticks or reservoirs for pathogens (**b**) and the remotely sensed information to produce the variables of the environmental niche (**c**). This allows calculation of the environmental niche for each species of host and tick (**d**) and the computation of an epidemiological network involving all reported associations between vertebrates, ticks, and pathogens (**e**), ranking the relative importance of each species in the network. The last step (**f**) involves evaluating the habitat overlap between hosts and ticks, the host availability for each species of tick, and adequate weighing according to derived relationships.

Figure 1 describes the workflow for compiling the necessary datasets. Most of the queries of public repositories were performed through scripts written in the R programming environment^11^, including downloads of remotely sensed information and coordinates for the distribution of hosts. Data on associated pairs of ticks, pathogens, and hosts were obtained from a literature search.

### Literature search of taxonomic associations among ticks, hosts, and transmitted pathogens in the Western Palearctic

Data on pairs of systematic associations among ticks, hosts, and pathogens in vertebrates and ticks were compiled from a literature review focused on the Western Palearctic (Fig. 1a). The region includes Europe, North Africa, northern and central parts of the Arabian Peninsula, and part of temperate Asia roughly to the Ural Mountains. The literature review was based on journals searchable in Thomson Reuters, Scopus, and PubMed, and used the scientific names of the genera of ticks as the keywords. A ‘record’ is a dual combination of either pathogen/tick/host combination at one site. If the same combination of partners was collected at the same site several times (e.g., through seasonal collections), it was included only once. However, the same combination of partners at different sites was accumulated to estimate the commonality of that association; the number of reports was used for weighting as described below. The literature review included reports published from 1970 to December 2014. As much as possible, the names of the pathogens were updated with results obtained by molecular approaches^10^. When not possible (i.e., the complex of *B. burgdorferi* species, which radically changed their names in the last 10 years), a generic name was included to reflect the relationships without relying on the current specific name of the pathogen.

The approach in data collection intends to be holistic, since it captures the long-term relationships of the partners in the network, but ignores other factors like abundance and seasonality of both ticks and hosts. Data on seasonality of hosts and ticks are scattered in the literature, resulting in an unreliable framework because sampling bias for large territories. Furthermore, the variables shaping the abundance and seasonality of hosts have a local character, changing drastically over short distances^7,8^, and are far to be well characterized over large scales of the territory. Both seasonality and abundance of the partners largely affect the recorded interactions^2^, including immune processes in the host, the ability of the tick to fill other biotic niches if a particular host species is absent, or the reservoir and transmission capacity of the host to the feeding ticks^2^. This is why we opted for an integrative view of the interactions between hosts, tick, and pathogens, that ignores these issues and provides a generalist view of the system. These issues warrant further elaboration on this framework that can be targeted in future research.

### Obtaining the coordinates of tick records from the literature data

Pairs of reliable coordinates of collections are necessary to evaluate the environmental niche of a tick species. We retrieved published coordinates for the collection of ticks^10^ updated with new data as explained above. Thus, the dataset on the distribution of ticks includes pairs of reliable coordinates, the third level administrative divisions of Europe (NUTS3, an official definition applying only to Europe), and provinces of non-European countries belonging to the Western Palearctic.

### Obtaining the coordinates of vertebrate host records from the global biodiversity information facility (GBIF)

Reliable coordinates of vertebrate host records are necessary to deriving the potential distributions of each species from predictive models. Therefore, a list of unique records (i.e., without overlapping coordinates) was obtained for each host species at 0.01° spatial resolution through the GBIF using the package dismo^12^ for the R programming environment (Fig. 1b). We obtained ~3,800,000 records with coordinates for a total 289 host species. Species with less than 100 records were excluded from further analysis. The dataset of hosts includes details on high taxonomic arrangements (i.e., family and order) aiming for completeness of information.

### Capturing the environmental traits that shape the distribution of ticks and hosts

Correlative modeling involves procedures that aim to understand how environmental covariates (or predictors) restrict a species to a given territory. There is a tendency to use interpolated climate datasets to identify the geographic range in which permanent populations of organisms may proliferate^13,14^. Interpolated climate datasets are prepared by compiling data from climate recording stations. These data retain enormous value for describing the climate of large territories. However, because these data are interpolated, monthly climate data series for temperature and rainfall suffer from statistical issues, such as autocorrelation, in which the use of consecutive monthly interpolated climate values produce over-performing models. In simple terms, these models cannot be trusted because algorithms dramatically fail at evaluating the environmental niche if the explanatory variables are autocorrelated^15^. The common procedure in these cases is simply to drop the affected variables from the model. Keeping autocorrelated variables inflates the model, and dropping variables that are correlated results in a loss of their potential in explaining the distribution of the organism. Therefore, exploring other sets of data that are not derived from interpolated information is desirable adequately capturing the environmental niche driving the distribution of organisms.

We used remotely sensed information on temperature and vegetation (Fig. 1c), retaining the most significant information after transformation by harmonic regression^16^. This technique is used to decompose a complex signal into a series of individual sine and cosine waves characterized by specific amplitudes and phase angles. The technique reduces the number of variables necessary to describe an event while retaining the complete information of the time series^16,17^. A variable number of components can be extracted, but generally only a few terms are necessary to describe annual, semi-annual, and smaller components of the seasonal variance. In summary, the harmonic regression produces coefficients that fit the seasonal behavior of each pixel in a series of images^17^. When the equation is filled with a time term, the coefficients reconstruct the pattern of the temporal variable to be measured. Thus, these coefficients are orthogonal and uncorrelated performing better than interpolated climate datasets for capturing the abiotic niche of an organism because they describe both the pattern (seasonal components) and ranges of the climate variables^16,17^.

A lineal regression has the form y=a+bx (where a and b are the coefficients of the regression), but in the harmonic regression the coefficients have sine and cosine transformations because they deal with time series with a periodic behavior. Therefore, a harmonic regression takes the following form:

y=a1+(a2*(SIN(2πt)))+(a3*(COS(2πt)))+(a4*(SIN(4πt)))+(a5*(COS(4πt))..).+…(an*(COS(xπt)))+(an*(SIN(xπt)))

In this regression, y is the temperature or Normalized Difference Vegetation Index (NDVI) at time t, and a is the designated coefficients. Though a large number of coefficients can be obtained, in practice four coefficients (plus the independent term, a1) are enough^7,16^. The first coefficient represents the mean value of the variable, whereas the rest of the coefficients describe the seasonality; the second and third coefficients shape the slope and the duration of the spring peak, and the fourth and fifth coefficients draw the slope and duration of the autumn curve^16,17^. The coefficients of the harmonic regression are hereafter referred to as ‘environmental covariates’ because they explicitly represent the environmental niche that may be occupied by an organism. The complete set of coefficients is available in the accompanying dataset (Data Citation 1).

We used the MODIS satellite data for land surface day temperature (LSTD), land surface night temperature (LSTN), and the NDVI. The latter is a measure of photosynthetic activity, which has been used as a proxy for vegetation stress and to derive water content at the vegetal strata, which is important for addressing the physiology of ticks^7,17^. The raw LSTD, LSTN, and NDVI data were obtained from the MODIS website (http://modis.gsfc.nasa.gov/data/dataprod, last accessed March 2015) at intervals of 16 days for the years 2002–2014 at a spatial resolution of 0.05°. After calculating monthly averaged values for LSTD, LSTN, and the NDVI over the complete period of 2002–2014, we obtained five coefficients for the harmonic regression of these time series. In the set of accompanying data, we included a script in R to load the remotely sensed information and produce and save the coefficients of the harmonic regression (Data Citation 1).

The resolution of the explanatory variables does not allow to capture the changes in the habitat introduced by i.e., human actions, like agricultural practices or urbanization. It also does not focus on the micro-environment, i.e., the conditions that shape the tick’s and host’s life cycles at the level of the vegetation. The scarcity and the local character of the available data addressing these micro-environment interactions, as well as the need to keep the framework tractable at a continental scale (i.e., with a balance between the total area and the resolution), precluded the inclusion of micro-environmental factors.

### Modelling the potential niche of the ticks and hosts

As our aim was to produce an overview of interactions between hosts and ticks to derive the probability of the circulation of a tick-transmitted pathogen, it was necessary to calculate how the occupancy of the environmental niche(s) by ticks and hosts overlaps (Fig. 1d). Probabilities of occurrence were produced using correlative modelling with the occurrences for each species and the set of environmental covariates. We used the pairs of coordinates for the reported distribution of every host species for each tick to identify regions where each species is predicted to occur to obtain the dimensions of the environmental niche. We independently modeled the presence of each species using the niche modelling algorithm MaxEnt integrated in the package dismo^12^ for R. This modelling algorithm was chosen because it demonstrated robust performance compared to other modelling algorithms when presence-only data is available. Models were developed with lineal and quadratic features, with a maximum of 10,000 background points, 10 replicates per species modelled, and 70% of points for training purposes, using cross-validation to compare the resulting models. The regularization multiplier was set to 1. Each model was replicated 100 times using the cross-validation function in MaxEnt to partition the data into replicate folds, with each fold used in turn to test the model. Maps of predicted niche occupancy were produced for each species of host and tick (Data Citation 1). We did not aim to project the results onto the geographic space, but to obtain the predicted niche occupancy for each species at intervals of the environmental covariates. This information was extracted from the maps using the package phyloclim^18^ for R.

### Building the network of interactions between ticks, hosts, and transmitted pathogens

To evaluate the biotic interactions between ticks, their hosts, and the transmitted pathogens, a network was built using data from the literature search (Fig. 1e). This network reflects the importance that each vertebrate has as a tick host or pathogen reservoir in the complete target region. The network is not a local index and is not related to the biological properties of the pathogens in the host or vector. The purpose was to obtain a general epidemiological framework based on previous network developments, which were revealed to be adequate for the purpose of representing these relationships^19^.

Networks exhibit system components (nodes) and the relationships between these components (links). Each node represents a species. The network is directed, as each edge links a pathogen ‘to’ a vertebrate or vector. The complete set of records was weighted according to reported methods^20^ before further analysis to avoid bias in sampling that could lead to misbalanced detection of specific pairs of partners. Several indices were used to measure network properties from which the relationships among hosts, ticks, and pathogens are derived. The degree centrality (DC) is the most basic measure of a network, representing the number of edges leaving (or arriving at) a given node after weighting by the total number of records containing this interaction. The DC provides an estimation of the strength of the association but does not evaluate the importance of each node in the context of the network. The node betweenness centrality (NBC) indicates how often a node is found on the shortest path between two nodes in the network^19,20^. The implicit meaning of the NBC in our application is the importance of a node in the flow of other components of the network and is considered a basic index defining the circulation of parasites or pathogens in an ecological network^20^. The PageRank (PR) is an index of centrality that assigns a universal rank to nodes based on the importance of the other nodes to which it is linked^19,20^. Therefore, the NBC and PR are complementary measures for capturing the importance of each node in the linkage of other nodes throughout the network^19^. These three indexes capture the biotic relationships between the interacting partners.

We built the epidemiological network for the set of species of ticks, reported hosts, and recorded pathogens in the target region from reports published from 1990 to 2014. Host-parasite data are sensitive to sampling effort. Consequently, the computation of individual centrality values is largely influenced by the intensity of sampling and reporting. To ensure that our findings are robust, we used an approach employed in similar studies^19,20^ for controlling variation in sampling, up-weighting the least sampled species and down-weighting the most sampled species. Specifically, we regressed the weight of each edge against the number of citations of the least sampled species (vertebrate, tick, pathogen) in each edge. This regression was highly significant (standardized beta=0.49±0.002, t=35.25, *P*<0.0001, R^2^=0.34; linear regression). We then additively rescaled the residuals to be greater than zero, replaced the original weights of the edges (number of parasites shared per pair of vertebrate species) with the rescaled residuals, and computed all centrality estimates using the package igraph^21^ for R. The dataset contains the complete list of interactions among ticks, hosts, and pathogen, necessary to build such an epidemiological network (Data Citation 1).

### Calculating the niche overlap between interacting partners

**Figure 2:**
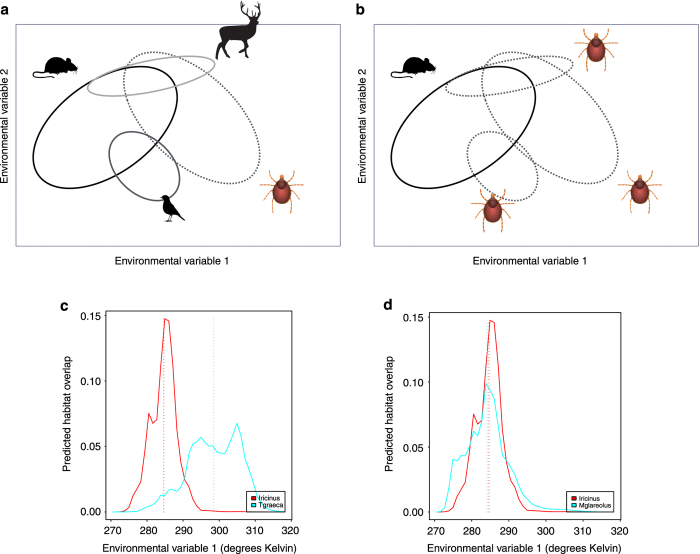
The structure of interactions between hosts and vectors in the environmental niche. The multivariate environmental niche is represented by only two variables (X and Y). The expected occurrence of ticks or hosts is represented by ellipses that account for the portions of the niche where permanent populations of the organisms can prevail. The intersection of these ellipses represent the portions of the environmental niche in which species can interact because they share parts of the niche. We represent two different kinds of interactions. (**a**) Several hosts interact with only one species of tick. (**b**) Several species of ticks interact with only one host species. The framework accounts for the niche overlap between suitable hosts and vectors and includes the importance of each host for the tick to evaluate the ‘strength’ of the interaction where niches overlap. (**c** and **d**) The niche utilization and overlap between the tick *Ixodes ricinus* and two reported hosts, *Testudo graeca* (**c**) and *Myodes glareolus* (**d**), in the dimension of the annual mean temperature with low niche overlap in the pictured dimension in (**c**). (**d**) Almost identical use of environmental resources by both the tick and that host. Broken lines indicate the median of the preferred niche occupancy.

The interaction likelihood between partners from two or more species is roughly proportional to the product of their relative abundance^22,23^. Niche occupancy by a species is not a proxy for species abundance, but it is a good indicator of the commonness of the species in the presence of a given combination of environmental variables. The niche overlap between a species of tick and each host is the amount of environmental niche shared between them. Calculating the niche overlap between pairs of interacting ticks and hosts is necessary to determine the extent of the interaction. Only those portions of the environmental niche shared by ticks and hosts are available for possible interactions between them. Figure 2 explains these concepts using two dimensions of the niche.

A few different indexes measure the niche overlap between two organisms; all of them take a list of expected occupancies at intervals of every environmental variable and compute the percent of habitat shared at each explanatory variable. We used the Schoener’s *D* distance^24^, which ranges from 0 (no overlap) to 1 (identical niches) to calculate the amount of niche shared in the dimensions of environmental covariates. This index measures the ‘availability’ of hosts at intervals of the multidimensional environmental niche based on the habitat overlap between the tick and each of its hosts. The Schoener’s *D* distance is regarded to be one of the most accurate techniques for measuring niche overlap from modelling results^24^ and is based on niche occupancy. Figure 2c,d illustrate two examples of niche overlap between the tick *Ixodes ricinus* and two of its hosts along one of the environmental variables, the average annual temperature. The figures show the occupancy by each species at intervals of the variable, as well as the median value of the variable occupied by the species, indicating the most commonly used portion of the niche. One of the hosts, *Testudo graeca*, is a species of turtle that uses relatively warmer portions of the niche, consequently sharing a small portion of the abiotic niche with the tick (Fig. 2c). The second species, *Myodes glareolus*, is one of the most widely reported hosts of this tick species^25^, and both the tick and host almost perfectly overlap in the available niche (Fig. 2d). As a proof of concept, the total niche overlap along the complete set of explanatory variables is 0.32 between *I. ricinus* and *T. graeca* and 0.85 between *I. ricinus* and *M. glareolus*.

### Calculating the probability of the occurrence of tick-transmitted pathogens in the environmental niche

The predicted niche occupancy (overlap) and epidemiological network describe the abiotic and biotic relationships between ticks and hosts, respectively. Thus, the predicted niche overlap of ticks and hosts (abiotic) can be weighted by the contribution of each host using the centrality indexes of the epidemiological network (biotic) to estimate the persistence of a pathogen in the dimensions of the niche. The procedure is a simple recursive weighting in which the contribution of every species of pathogen reservoir depends not only on the amount of niche overlap, but also its relative importance in the complete network of ecological relationships.

To quantify the probability of the occurrence of a pathogen in the environmental niche, we developed a metric called Centrality-weighted Habitat Overlap (CwHO), which is defined as:

$$\sum_{i=1}^{n}\left({[}^{D}T\theta H_{i}]*\partial_{TH}*\partial_{PH}\right)$$

where *T*=tick, *H*=host, *P*=pathogen, and *i*=correlative species of host in which the tick has been recorded or the pathogen has been detected. The equation reads ‘the sum of the habitat overlap between the focal tick and every host as measured by the Schoener’s *D* metric multiplied by the centrality of that host for the circulation of the tick and the centrality of that host for the circulation of the pathogen’. Both centrality values for each host are obtained from the product of the DC multiplied by the NBC derived from the epidemiological network explained above. It is implicit that the probability of pathogen occurrence (*∂*_*PH*_) cannot be computed without previous computation of the tick occurrence (*∂T*_*H*_).

**Figure 3:**
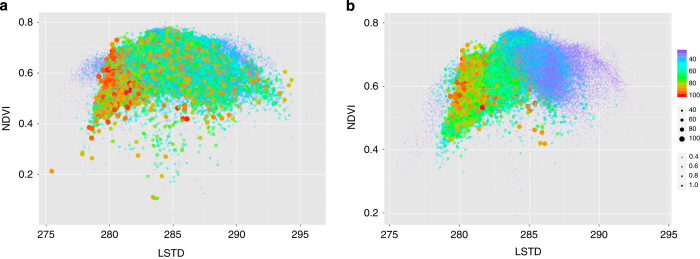
The plot of the predicted niche occupancy for the tick *Ixodes ricinus* calculated on the dimensions of average ground temperature (LSTD, in Kelvins) and average vegetation (NDVI, no unit). The plot indicates the intervals of the niche in which the tick can find suitable conditions for persistence considering only its abiotic component (**a**) or including the niche overlap with its reported hosts (**b**): the sum of the products of the habitat overlap of each host-tick pair multiplied by the centrality of each host in the epidemiological network and rescaled to the 0–100 interval to allow comparisons. For both panels, the predicted niche occurrence of the tick is indicated by the size, color, and transparency of the dot, combined. Transparency has been included to improve readability because each plot has more than 1.5 million dots.

**Figure 4:**
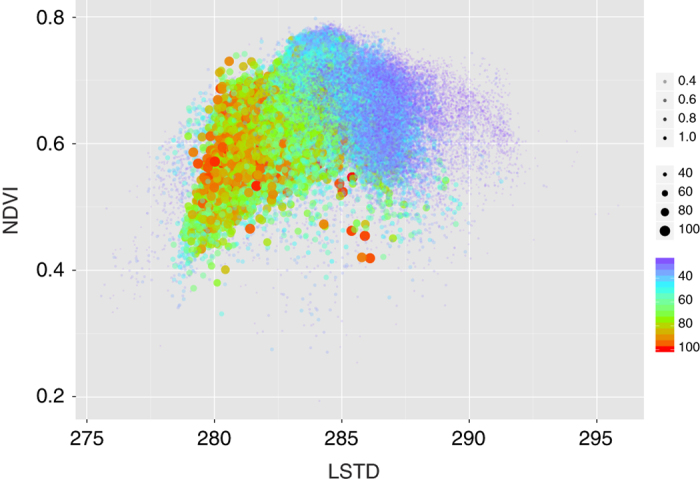
The plot of the predicted niche occupancy for the *Borrelia burgdorferi* complex obtained in the dimensions of the average ground temperature (LSTD, in Kelvins) and average vegetation (NDVI, no unit). The plot was obtained according to the evaluation of environmental suitability for the tick vector, habitat overlap with its reported hosts, and relative importance of each host in supporting the circulation of borreliae as obtained from an epidemiological network evaluating the importance of each reservoir. For both panels, the predicted niche occurrence of the pathogen is indicated by the size, color, and transparency of the dot, combined. Transparency has been included to improve readability.

The dataset was intended to compute this index for every tick and tick-transmitted pathogen for which the published reports provided a record of the relationships between tick, hosts, and pathogens. Here, we provide examples of calculations of CwHO for the prominent tick *I. ricinus* and the *B. burgdorferi* pathogens, which are transmitted by this tick and supported by a wide array of reservoirs^25^. Figure 3a is a plot of the niche occupancy of *I. ricinus* in its abiotic component as calculated on the two dimensions of LSTD and the NDVI. Figure 3b shows the same results for when the abiotic suitability was weighed by the biotic component of the tick: the sum of the products of the habitat overlap of each host-tick pair multiplied by the centrality of each host in the epidemiological network (rescaled to the 0–100 interval). Therefore, the tick was restricted to a narrower interval of the abiotic niche, as outlined by the combined preferences of its hosts and their relative roles in the circulation of the tick populations. Complete scripts for calculations and plots were included in the accompanying dataset (Data Citation 1). Figure 4 shows the probability of occurrence of the bacteria in the group *B. burgdorferi* sensu lato in the same dimensions of the niche. The plot was obtained by further weighing the results in Fig. 3b by the centrality values of the reservoirs. Inclusion of host centrality for the pathogen produced only slightly different results than those obtained for the tick alone. The automatic translation is that the pathogen should exist wherever the tick is present, as a large array of vertebrates serve as reservoir for the pathogen. Existing reports and meta-analyses of the distribution of *B. burgdorferi* in Europe show that the pathogens are found everywhere the vector exists^26^.

**Figure 5:**
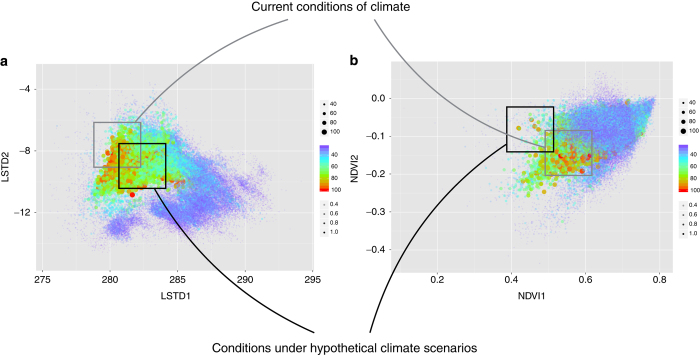
An example of the performance of the proposed framework to track the impact of climate trends on the expected occurrence of pathogens transmitted by ticks when the expected niche occupancy is plotted against the environmental niche instead of the geographic dimensions. The plots show the expected niche occupancy of the *Borrelia* pathogens in two dimensions of temperature (**a**) and two dimensions of the Normalized Difference Vegetation Index (NDVI) (**b**). The first dimension is the annual average temperature (LSTD_1_) or NDVI (NDVI_1_). The second dimension is the slope of the spring time temperature (LSTD2) or NDVI (NDVI_2_). Gray squares in both plots assume a set of hypothetical current conditions under which the pathogen could find a range of values of expected occurrence. A hypothetical climate shift, marked by a black square in the figure, would ‘move’ the environmental conditions to a different position. Because organisms track their niche, the predicted occupancy would shift and the change is easy to track.

Figure 5 schematically illustrates further application of this framework, which allows an explicit evaluation of the persistence of a tick or pathogen under different combinations of abiotic conditions, such as two variables of temperature (Fig. 5a) or two variables of the NDVI (Fig. 5b). This framework also allows an estimation of the possible impact of the climate trends on the niche occupancy by ticks and their hosts, assuming that both hosts and ticks track their niche, as has been reported repeatedly^15^. Figure 5 also shows a hypothetical scenario in which the average annual temperature is warmer (LSTD1) and the temperature increases faster in the spring (negative values for LSTD2, accounting for the slope of the time series), as well as sparser and drier vegetation (NDVI1) and a slow increase in vegetation greening in the spring. The schematic displays a poorer set of conditions for the persistence of *I. ricinus* under the hypothetical scenario of climate change, an estimation that can be quantitatively measured using the proposed CwHO.

### Code availability

Scripts to i) select the climate MODIS images and produce the coefficients of the harmonic regression, ii) obtain seasonal disaggregation from the coefficients, and iii) perform the calculations of CwHO are available in the accompanying dataset (Data Citation 1). The scripts were developed in R version 3.2.3, and using the associated libraries as indicated in the scripts. There are no restrictions to use the provided code.

### Possible gaps and caveats of the methodology

We aimed to reconcile the abiotic and biotic aspects of the ecosystem that delineate the forces operating on tick-transmitted pathogens, proposing a methodology to evaluate the niche dimensions of tick-transmitted pathogens. The relative contribution of the hosts to the circulation of pathogens is derived from the centrality values of an epidemiological network, which is a radically new approach for exploring the many facets of tick-borne pathogens rather than geographic risk analysis^19^. Reservoir capacity describes the absolute contribution made by a particular reservoir host species to the natural prevalence of infection by a given pathogen within a certain site and is dependent on its infectivity for feeding ticks and the duration of the infective period^27–29^. Thus, on local or regional scales, relative host abundance rates are critically important for describing the activity of foci of tick-borne pathogens. The compilation of a continental dataset of host preferences by ticks cannot describe the subtle local processes that impact the reservoir capacity of hosts, because a tick can be perceived as generalist or specialist according to the geographical scale. On the community scale, stochastic mechanisms and tolerance of ecological conditions can modify host diversity, the abundance of ticks on each host species, and the prevalence of pathogens in ticks^7^. As the mechanisms driving stochasticity in host preferences by ticks are still undefined, the distribution of host species across communities may facilitate or impede the distribution of tick-transmitted pathogens on the local scale, unbalancing the proportion of ticks feeding on hosts with or without reservoir capacity. Recent reports in the field related the physiological traits of the hosts with their ability to reservoir and transmit tick-borne pathogens^29^. These are promising meta-analyses of the reservoir and transmission capacities of several species of hosts that cannot yet be accommodated into a general hypothesis. Thus, our approach considers the epidemiological network as an object that describes the semantics of the interactions at the holistic level and cannot cope with local stochasticity.

Possible drawbacks in our methodology can affect the estimation of the contact rates between ticks and hosts through calculation of niche overlap, again because this development cannot handle the local stochastic processes. When hosts and ticks have the chance to meet because they share a portion of the abiotic niche, whether they will interact is widely thought to be the product of an array of behavioral and phenotypic aspects^30^. The interaction is prevented if both partners do not share a portion of the environmental niche or if they have not the chance to meet. From this perspective, the interactions can be mapped onto networks that accordingly vary along with variation in the environmental traits. One potential drawback to a direct approach to niche overlap is that it still assumes that the observation of any interaction is only a function of the environmental variables and co-occurrences of species without accounting for the actual biological importance of that interaction.

### Prospects of future research

It has not escaped to our attention that the postulated framework could be applied to further capture the relative importance of each host-tick pair in the circulation of pathogens. The removal of single nodes representing hosts could provide an insight into the particular contribution of the node, assuming a re-distribution of the ticks affecting the removed host to other available hosts in a way proportional to the preferences derived from the structure of the network. The changes in the epidemiological network could then be partly extrapolated by removing/including specific pairs of interacting ticks and hosts and comparing the centrality values of the network and the Centrality-weighted Habitat Overlap under the gradient of environmental variables. This could result in the capture of the already reported and still unaddressed variable epidemiological relationships at different portions of the environmental niche^9,19,23,26,27^.

The framework can capture the impact of particular species of hosts in both the resilience of the network to collapse and the spread of pathogens: most central hosts are considered super-spreaders^9,20,27,28,31,32^. Its identification through the network, its restriction to particular portions of the environmental niche, and its contribution to the circulation of pathogens at different portions of that niche, are key features to unravel the core mechanisms that drive the networks of tick-transmitted pathogens. The key idea is that, despite the complexity of the underlying epidemiological processes, and the multiplicity of routes that tick-transmitted pathogens can follow because the interactions with different hosts, the dynamics of the processes are dominated by a set of most important connections between partners. As mentioned before, seasonality could be incorporated in these constructs, and study how relationships and centrality values between interacting partners change along a time gradient. Currently, these analyses could only be focused on regional scale studies because the scarcity of available data.

This study intended to develop a coherent approach to dealing with the study of tick-transmitted pathogens in the environmental niche. Notably, interactions between ticks and pathogens have classically been evaluated strictly in the geographic dimensions, which cannot handle the set of conditions that operate on these associations. This framework highlights the benefit of integrative approaches based on the consideration of both biotic and abiotic traits instead of perceptions derived from observations of single pairs of partners. To improve the future development of these models across spatial scales, we call for accelerated collection of spatially and temporally explicit species data. Ideally, these data should be sampled to reflect variation in the underlying environment across large spatial extents and at fine spatial resolution.

## Data Records

The data obtained and curated as explained above, as well as the interactions identified in the epidemiological network, are available at datadryad.org (Data Citation 1). Table 1 documents the contents and format for the complete dataset.

## Technical Validation

### Validation of data completeness for ticks, hosts, and pathogens

It was evident at initial stages of the bibliographic search that the use of a long series of keywords would miss papers that fit the criteria of the search. In other words, the submission of a search chain based on the scientific names of the ticks AND the scientific names of the *possible* hosts, AND every country of Europe would lack i) specific information for some species of ticks or hosts not previously recognized in the target territory, ii) newly recorded pathogens (previously found in other regions of the world), and iii) names of countries that changed in recent years. Therefore, we preferred to perform a deliberately relaxed query including only the scientific names of the genera of the ticks, and to keep only the papers dealing with ecological information after a critical evaluation of the abstract. Papers devoted to the clinical presentation, treatment, or diagnosis of the disease were included in the dataset only if adequate information was available about the host, tick, and/or pathogen(s). We are aware that the so-called ‘grey literature’ (abstracts to scientific meetings, poster presentations, etc.) are poorly represented in the compiled dataset. However, we are confident that most of these presentations are commonly published later in searchable journals. Duplicated papers obtained from the three sources were removed. We purposely removed every report concerning livestock or humans because they are recognized as accidental hosts, generating spurious information that distorts the natural relationships in the community of tick-borne pathogens^19^.

### Validation of specific names of ticks and pathogens and disambiguation of tick-host-pathogen interactions

A set of rules was established to remove unreliable information: i) records lacking the specific determination of the pathogen or the tick were not included, leading to the exclusion of serological data; ii) every organism reported as detected in ticks while feeding on hosts via molecular analysis was rejected because molecular techniques applied to feeding ticks probably detect nucleic acids in remnants of the blood ingested by the ticks; iii) data from humans were not included because they are considered accidental findings; and iv) the study is restricted to ticks of the family Ixodidae in the target region because of the lack of agreement among experts regarding the correct taxonomy of the family Argasidae^33^. Specific names of ticks were checked against the consensus list of valid species of ticks in the family Ixodidae^34^. In a further effort to avoid issues in the identification of pathogens, only data on ticks-pathogens or hosts-pathogens published after the year 1990 were included in the dataset. Data regarding associations of the ticks, pathogens, and livestock were purposely removed from the dataset, as data from livestock distort the real dimensions of the network^12^.

### Host disambiguation

Records lacking specific determination of the host were not included. In the 87.3% of published reports, the scientific name of the host was already included. The set of scientific names was updated using the services of the Open Tree of Life (OTL) using the package rOTL^35^ for R to obtain the accepted official name of the vertebrate. Hosts reported by their common (not Latin) names were checked against the GBIF database. The Sixty-one percent of common names reported in the literature search were unambiguously resolved to their scientific names.

### Location disambiguation

Coordinates were provided in 63.8% of the examined papers to a precision of two decimal places. All of the coordinates were transformed to latitude/longitude, plate carré projection, with a precision of two decimal places. Data were reported with reference to the name of a locality and the country in 22.4% of cases, which were then translated to pairs of coordinates using geolocation services if names of localities were unambiguously resolved. The remaining 13.8% of reports were referred to administrative units of variable size in the target territory. This information was made available in the final dataset, but these records were not used to build the niche preferences of the species because they refer to territories of variable size and not to pairs of coordinates from which information on the environmental niche cannot be extracted.

### Detection of ambiguous pixels in satellite-recorded data

Remotely sensed imagery may have gaps in regions near the poles of the Earth because of the long-lasting accumulation of snow, ice, or clouds. The effects are larger in the target region because of the proximity to the North Pole. The detection of these gaps and the filling with estimated values may be unreliable if the number of consecutive gaps is too high. We produced cubic splines to fit the maximum number of gaps in these regions. However, some regions in the far north were not included in the final set of images because they were covered by snow, clouds, or ice for periods longer than 4 months. Distorted values affected by these problems were replaced by ‘not available’ in the images.

## Additional Information

**How to cite this article:** Estrada-Peña, A. & de la Fuente, J. Species interactions in occurrence data for a community of tick-transmitted pathogens. *Sci. Data* 3:160056 doi: 10.1038/sdata.2016.56 (2016).

## Supplementary Material



## Figures and Tables

**Table 1:** Summary of the complete dataset generated and described in this study.

**Name of the file or folder**	**Time span for which information was collected**	**Number of files**	**Available format**	**‘Readme’ file with short descriptions of the contents**	**Number of species of ticks (genera)**	**Number of species of hosts (genera, families)**	**Number of species of pathogens (genera)**	**Number of records**	**Structure of the tables**
Folder ‘Environmental variables’	2002–2014	15 (plus two scripts in R)	Raster files in .asc format	Yes (including metadata on coordinates and projection)	NA	NA	NA	NA	NA
Coordinates of ticks with hosts records	1970–2014	1	Table in .csv format	Yes	48 (8)	NA	NA	6230 (5630 georeferenced)	column 1: species of tickcolumn 2: genus of tickcolumn 3: species of hostcolumn 4: genus of hostcolumn 5: family of hostcolumn 6: order of hostcolumn 7: country of reportingcolumn 8: administrative division of reporting (NUTS)column 9: geographical latitudecolumn 10: geographical longitude
Coordinates of Hosts	Unknown. As available in GBIF	1	Table in .csv format	Yes	NA	245 (146, 68)	NA	3837362	NA
Folder ‘Maps of predicted distribution of hosts’	2002–2014 (for explanatory variables)	230	Raster files in .asc format	Yes (including metadata on coordinates and projection)	NA	230	NA	NA	NA
Folder ‘Maps of predicted distribution of ticks’	2002–2014 (for explanatory variables)	108	Raster files in .asc format	Yes (including metadata on coordinates and projection)	8 (5)	NA	NA	NA	column 1: species of hostcolumn 2: genus of hostcolumn 3: family of hostcolumn 4: order of hostcolumn 5: geographical latitudecolumn 6: geographical longitude
Dataset of interactions between ticks-hosts and pathogens	1990–2015	1	Table in .csv format	Yes	50 (7)	466 (87, 26)	68 (16)	13129	column 1: species of ‘cargo’ (tick or pathogen)column 2: genus of ‘cargo’column 3: species of ‘carrier’ (vertebrate or tick)column 4: genus of ‘carrier’column 5: family of carrier (if vertebrate)column 6: order of carrier (if vertebrate)
Calculating CwHO	NA	1	Script in R (example)	Yes	NA	NA	NA	NA	NA
The table indicates the name of each file or folder in the dataset, the time span for which the information was obtained, and the number of files in each folder. Additional details are provided for the number of species and genera of ticks, hosts and pathogens available in each file (also families in the case of hosts), the total number of records available and the structure of the tables (column names) in the files in.csv format.									
